# Beyond Bronchopulmonary Dysplasia: A Comprehensive Review of Chronic Lung Diseases in Neonates

**DOI:** 10.7759/cureus.64804

**Published:** 2024-07-18

**Authors:** Khaled El-Atawi, Muzafar Gani Abdul Wahab, Jubara Alallah, Mohammed F Osman, Moustafa Hassan, Zohra Siwji, Maysa Saleh

**Affiliations:** 1 Pediatrics, Latifa Women and Children Hospital, Dubai, ARE; 2 Pediatrics, McMaster University, Hamilton, CAN; 3 Neonatology, King Saud Bin Abdulaziz University for Health Sciences, Jeddah, SAU; 4 Neonatology, King Abdulaziz Medical City, Ministry of National Guard - Health Affairs, Jeddah, SAU; 5 Neonatology, Tawam Hospital, Al Ain, ARE; 6 Neonatology, Dubai Hospital, Dubai, ARE; 7 Pediatrics and Child Health, Al Jalila Children's Specialty Hospital, Dubai, ARE

**Keywords:** neonates, pulmonary hypoplasia, congenital diaphragmatic hernia (cdh), chronic lung diseases in neonates, bronchopulmonary dysplasia (bpd)

## Abstract

In neonates, pulmonary diseases such as bronchopulmonary dysplasia and other chronic lung diseases (CLDs) pose significant challenges due to their complexity and high degree of morbidity and mortality.

This review discusses the etiology, pathophysiology, clinical presentation, and diagnostic criteria for these conditions, as well as current management strategies. The review also highlights recent advancements in understanding the pathophysiology of these diseases and evolving strategies for their management, including gene therapy and stem cell treatments.

We emphasize how supportive care is useful in managing these diseases and underscore the importance of a multidisciplinary approach. Notably, we discuss the emerging role of personalized medicine, enabled by advances in genomics and precision therapeutics, in tailoring therapy according to an individual's genetic, biochemical, and lifestyle factors.

We conclude with a discussion on future directions in research and treatment, emphasizing the importance of furthering our understanding of these conditions, improving diagnostic criteria, and exploring targeted treatment modalities.

The review underscores the need for multicentric and longitudinal studies to improve preventative strategies and better understand long-term outcomes. Ultimately, a comprehensive, innovative, and patient-centered approach can enhance the quality of care and outcomes for neonates with CLDs.

## Introduction and background

Chronic lung diseases (CLDs) in neonates encompass a range of conditions such as bronchopulmonary dysplasia (BPD), pulmonary hypoplasia, congenital diaphragmatic hernia (CDH), congenital pulmonary airway malformation (CPAM), pulmonary interstitial glycogenosis (PIG), neuroendocrine cell hyperplasia of infancy (NEHI), and alveolar capillary dysplasia with misalignment of pulmonary veins (ACD/MPV). CLDs represent persistent and significant clinical challenges in neonatology [1]. These multifactorial disorders often occur in premature neonates, necessitating mechanical ventilation and contributing considerably to neonatal morbidity and mortality [2]. Thus, this subject is of significant importance to researchers, clinicians, and healthcare providers caring for this vulnerable population [3,4]. The primary objective of this review is to present an updated and comprehensive overview of CLDs in neonates.

CLD in neonates is traditionally associated with BPD, a condition originally described in 1967 by Northway et al. after the advent of positive-pressure ventilation [5]. As advances in perinatal medicine improve survival rates among extremely preterm infants, other CLDs have become apparent, broadening the scope and complexity of this issue. Moreover, despite advancements in neonatal intensive care, CLD incidence rates have remained relatively stable [6]. Further research is needed to understand the etiologies and long-term implications of CLD, including complications and prognostic factors, to advance diagnostic capabilities, optimize management strategies, and develop preventative measures.

The primary objective of this review is to present an updated and comprehensive overview of CLDs in neonates. We aim to evaluate the current literature and discuss the etiology, pathophysiology, clinical presentation, and diagnosis of these diseases, along with the latest strategies for management and prevention. This review also assesses the complications, prognoses, and measures for improving outcomes of these conditions.

## Review

BPD

BPD is associated with inflammation, lung injury, genetic predisposition, and impaired lung development. It is a complex disease with multiple contributing factors influenced by the timing and exposure duration. The initial definition, which stipulated the need for oxygen at 28 days and radiological evidence of changes, was amended to specify the need for oxygen therapy at 36 weeks (corrected gestational age) [7,8]. None of these criteria addressed the diverse clinical practices and disease scope, however. Therefore, in 2000, the National Institute of Child Health and Human Development proposed a new definition of BPD as "a condition affecting infants born less than 32 weeks of gestation who require supplemental oxygen for at least 28 days and at 36 weeks postmenstrual age" [9]. The definition also includes stratification of the disease based on the severity of the oxygen requirement and type of respiratory support at 36 weeks postmenstrual age. Unlike previous definitions, the current guidelines do not require radiographic changes for diagnosis. Further standardization of the definition of BPD, including its “physiologic” criteria, is needed, as argued by Walsh et al. [8]. Differentiating the diverse phenotypes of severe BPD also has been suggested as a further step in categorizing these cases. The occurrence of BPD in infants up to 28 weeks of gestational age has remained constant at around 40% over recent years, or about 10,000 to 15,000 new cases annually in the United States [10-13]. The interpretation of these statistics, however, is complicated due to changes in definitions and oxygen therapy approaches.

Despite advancements (e.g., routine application of antenatal steroids for threatened preterm birth, surfactant use for respiratory distress syndrome), rates of BPD persist, primarily due to the lack of effective treatments for preventing neonatal lung injury and chronic disease [14,15]. Its prevalence in mechanically ventilated infants is inversely proportional to their gestational age and birth weight, indicating a link between incomplete lung development and BPD [16]. Disease development may be further influenced by mechanical ventilation, oxygen toxicity, infections before and after birth, inflammation, growth restriction, and nutritional deficits, as well as genetic predisposition [17]. Infants born prematurely and small for their age or with intrauterine growth restriction face higher odds of adverse pulmonary outcomes, including BPD, compared to other infants [18]. Insufficient nutrition in the first week after birth also has been linked to BPD [19]. Intriguingly, exclusive use of breast milk has been associated with a decreased risk of BPD [20].

Exposure to high concentrations of oxygen also has been found to trigger a BPD-like condition, suggesting that restricting oxygen use or targeting lower saturation could reduce BPD rates [21,22]. Premature infants' lungs are particularly vulnerable to oxidative stress, leading to alveolar cell injury [23,24]. Short-term exposure to high oxygen levels can cause long-term lung changes, and early life exposure to high oxygen concentrations has been linked to increased BPD risk [25,26].

BPD is mainly found in preterm infants who have received positive pressure ventilation, suggesting an important role of mechanical trauma in BPD's pathophysiology [27]. The premature lungs' need for ventilatory support at birth, the difficulty in maintaining functional residual capacity due to surfactant deficiency, and non-uniform lung expansion leading to overdistension and atelectasis may all contribute to BPD. Mechanical ventilation can further cause alveolar over-inflation, potentially exacerbating prenatal inflammation-induced injuries [6,28].

The impact of prenatal inflammation and chorioamnionitis on BPD risk is a matter of debate [29]. Although some studies point to the risk of lung injury and impaired alveolarization [30,31], a meta-analysis of numerous studies has found little association between chorioamnionitis and BPD after adjusting for gestational age [32]. Postnatal inflammation and hospital-acquired infections, however, are widely accepted as contributing factors to BPD [33]. Elevated pro-inflammatory cytokines in premature infants, such as TNFα, IL-8, IL-1β, and IL-6, also are associated with increased risk of BPD [34-36].

In addition to these environmental factors, studies on twins have suggested a significant genetic component, with genetic and shared environmental factors accounting for up to 65% of BPD susceptibility variances [37,38]. Genome-wide association studies have been conducted to identify potential genetic markers associated with BPD, but results thus far have been inconclusive [39]. Another smaller analysis concluded that the SPOCK2 gene may represent a possible candidate susceptibility gene and a key regulator of alveolarization [40].

Clinical Features and Diagnosis of BPD

On physical examination, BPD symptoms in infants are disparate, ranging from tachypnea to severe retractions and, occasionally, intermittent expiratory wheezing. Similarly, the evolution of BPD is observable in chest radiographs, with manifestations shifting from clear lung fields to diffuse haziness and coarse interstitial patterns, indicating atelectasis, inflammation, or pulmonary edema. More severe BPD is evident in hypoxemic and hypercapnic patients requiring mechanical ventilation and oxygen supplementation with decreased tidal volume, increased airway and vascular resistance, decreased dynamic lung compliance, and uneven airway obstruction leading to gas trapping and hyperinflation [41]. The disease course can culminate in bronchomalacia, causing airway collapse during expiration. A prospective study categorized infants with severe BPD (median postmenstrual age of 52 weeks) into one of three distinct phenotypes: obstructive, mixed, or restrictive [42]. Frequently, these patients experience increased pulmonary vascular resistance due to disruption of pulmonary vascular growth and reduced cross-sectional area of pulmonary vessels, culminating in pulmonary hypertension [43].

The diagnosis of BPD is contingent upon the criteria set forth in a standardized definition, such as that proposed by the National Institute of Child Health and Human Development, which considers factors like the need for oxygen supplementation, gestational age, postmenstrual age, and disease severity. The clinical diagnosis is predominantly determined by the need for oxygen supplementation at 36 weeks postmenstrual age as it is the most practicable approach. To confirm the diagnosis, a physiologic oxygen reduction test may be conducted. Infants are classified as having BPD if their oxygen saturation level drops below 90% within a 60-minute period after exposure to room air (correcting for altitude as appropriate) [8].

Management of BPD

Early management of BPD is multifaceted and involves the use of supplemental oxygen, early respiratory support, surfactant administration, invasive mechanical ventilation, caffeine, postnatal steroids, diuretic therapy, and nutritional strategies. Studies have shown that resuscitation of term infants using FiO2 0.21 can produce favorable outcomes, but the results were inconclusive for preterm infants [44,45]. Moreover, the results of a meta-analysis showed no benefit to starting resuscitation with FiO2 0.3 in comparison to FiO2 0.6 in preventing BPD [46]. Some evidence has linked hypoxia to a higher risk of death; thus, immediately starting continuous oxygen saturation monitoring after birth and adjusting supplementary oxygen levels to reach a SpO2 measurement above 80% during the first five minutes of life is the only current recommendation [47].

The risk of mortality prior to discharge increases when oxygen saturation falls below 85% to 89% [48]. However, no correlation has been identified between saturation of 91-95% and a higher incidence of “physiological” BPD; thus, it is recommended to establish saturation targets between 90% and 95% for newborns who need supplemental oxygen [49]. Overall, the optimal oxygen supplementation strategy for preterm infants remains an area of ongoing research and debate, and individual patient monitoring and tailored oxygen therapy are crucial for achieving the best outcomes.

Sustained inflations at birth in preterm neonates who need delivery room resuscitation have not been shown to reduce the risk of BPD [49,50]. Exogenous surfactant treatment also failed to significantly reduce the prevalence of BPD in three significant randomized controlled trials (RCTs) comparing prophylactic surfactant therapy with early continuous positive airway pressure (CPAP) [51,52]. Other meta-analysis data have not indicated any advantage of using the intubation-surfactant-extubation (INSURE) method over initiating CPAP [53]. Alternative techniques include minimally invasive surfactant treatment (MIST) and less invasive surfactant administration (LISA), where the surfactant is delivered during spontaneous breathing on nasal CPAP [54]. However, the largest multicenter RCT examining children with extremely low birth weight who received LISA versus endotracheal surfactant did not reveal a significant decrease in the incidence of BPD [55].

Findings from RCTs and meta-analyses support the early introduction of CPAP for infants at risk of BPD in the delivery room, with meta-analyses showing a slight but significant decrease in the incidence of BPD for those first supported with CPAP [56,57]. A flow driver, ventilator-driven CPAP, and “bubble CPAP” are some of the methods used to generate CPAP [56]. A meta-analysis of data from several studies revealed that bubble CPAP had lower failure rates than a ventilator or flow driver-regulated CPAP, but more data are needed to support the superiority of one modality over another, as another study showed that using a bubble CPAP did not correspond to a lower incidence of BPD [58]. Another technique, known as nasal intermittent positive pressure ventilation, promotes respiratory stability by using short pressure increases above the level of nasal CPAP support [59]. Although studies have demonstrated that nasal intermittent positive pressure ventilation in preterm infants can decrease the need for intubation, a lower risk of BPD has not been observed [60,61].

The current body of evidence recommends considering volume-targeted ventilation strategies when invasive mechanical ventilation is unavoidable. A comprehensive meta-analysis demonstrated a notable reduction in the combined risk of death or BPD at 36 weeks in cases using volume-targeted ventilation [62]. However, the primary use of high-frequency ventilation has not been uniformly effective in decreasing BPD in various RCTs, and it did not significantly enhance lung function when assessed between the ages of 16 and 19 years [63,64]. As such, the routine use of elective high-frequency ventilation in preterm infants at risk of BPD is not recommended.

A consistent association has been observed between the necessity for invasive ventilation at day of life (DOL) 7 and an increased risk for BPD [65]. One study revealed a significant rise in BPD for extremely low-birth-weight infants younger than 28 weeks gestational age if extubation occurs after DOL 8, compared to extubation between DOL 1 and 3-8 [66]. Another study found that the total number of days that positive pressure was administered through an endotracheal tube was a better predictor of unfavorable long-term pulmonary outcomes, compared to the number of invasive mechanical ventilation courses [67]. Early extubation, even in the presence of a potential need for reintubation, seems to contribute to improved pulmonary outcomes and a shorter hospital stay [68]. Invasive mechanical ventilation within 48 hours of the first extubation attempt is significantly associated with the combined outcome of BPD or death but not with an increased risk for moderate to severe BPD alone [69]. These data support proactive weaning of invasive mechanical ventilation during the first week of life, as well as an extubation trial in newborns who can handle weaning to low settings.

Caffeine citrate within the first three days of life greatly lowers the incidence of BPD and associated long-term neurological morbidity [69,70]. An RCT demonstrated that initiating caffeine within 10 days of life led to a substantial decrease in the occurrence of BPD (adjusted odds ratio = 0.63, P < 0.001) [71]. In older children (age 11) with previous BPD, those who received caffeine showed notable improvement in expiratory flow [72]. Administering caffeine to infants within the first three days of life can have a significant effect [72,73], although the dose and timing remain a matter of debate. An ongoing RCT (NCT03086473) is investigating the impact of early (two hours of life) versus late (12 hours of life) administration of caffeine.

Regarding the usefulness of postnatal steroids to prevent and treat BPD, early administration of dexamethasone can reduce the duration of mechanical ventilation and the incidence of BPD, but it is linked to an increased risk of neurodevelopmental impairment and cerebral palsy and thus should not be used in the first week of life [74]. Beyond the first week, dexamethasone may help reduce the occurrence of neurodevelopmental impairment in infants at high risk for poor pulmonary outcomes [74]. In infants older than one week who are on mechanical ventilation, a low-dose course of dexamethasone has been shown to improve the rate of extubation at the conclusion of the treatment without lowering the risk of BPD [75]. Further evaluations showed no negative effects or advantages in neurodevelopment [76]. Therefore, the use of postnatal dexamethasone should be restricted to infants most at risk for BPD who continue to require mechanical breathing after 21 days of age [77]. Low-dose hydrocortisone therapy has been linked to a considerable rise in BPD-free survival and a reduced prevalence of the condition, particularly in infants who had chorioamnionitis [78-80]. Early inhaled budesonide has shown promise in reducing the occurrence of BPD in an RCT involving 437 infants who received budesonide and 417 who received a placebo [81]. However, this treatment also has been associated with an increased risk of mortality and thus is not recommended for BPD prevention [82].

Diuretic therapy is commonly used in preterm infants with evolving and established BPD, especially infants with higher levels of respiratory support. Loop and thiazide diuretics are often employed to manage chronic, mild pulmonary edema in the “New BPD” phenotype [83]. However, the impact of diuretic initiation on respiratory status improvement has not been confirmed [83,84]. A multicenter, retrospective, cohort study found a significant association between longer exposure to furosemide and reduced incidence of BPD, but causality cannot be inferred due to the study design [85]. Diuretics can lead to metabolic bone disease, nephrocalcinosis, electrolyte loss, and poor weight gain and so their use should be carefully weighed against these side effects [85,86].

Nutritional strategies play a crucial role in managing BPD. Adequate postnatal nutrition is essential for maintaining lung growth and repair. A retrospective cohort study revealed a significant association between lower energy intake during the first four weeks of life, increased fluid intake, and the occurrence of BPD [87]. Hence, strategies such as relative fluid restriction, early introduction of enteral feedings, and optimized parenteral nutrition components should be considered [88]. In terms of milk feeding, an RCT demonstrated a significant reduction in BPD incidence in infants who received donor milk, compared to the formula-supplemented group [89]. Additionally, exclusive feeding with fresh maternal breast milk was associated with a decrease in BPD [90]. Therefore, maintaining an exclusive human milk diet, preferably using fresh maternal breast milk, is recommended in the management of infants with early and evolving BPD.

The discovery of the role of growth factors such as vascular endothelial growth factor and transforming growth factor-beta has highlighted the importance of angiogenesis in alveolarization, offering potential targets for future therapeutic interventions [91]. Further, an exploration into epigenetic modifications has revealed potential markers for BPD to improve early detection and preventative strategies [92].

Pulmonary hypoplasia

Pulmonary hypoplasia, characterized by underdeveloped lungs, is a significant concern in neonates and a leading cause of neonatal mortality and morbidity [93]. The incidence rate for pulmonary hypoplasia is about 1.4 per 1,000 for all births and between 0.9 and 1.1 per 1,000 for live births [94,95], although these figures may be underreported as milder cases are often identified later when respiratory symptoms appear. Various antenatal factors contribute to its development, including CDH, oligohydramnios, and certain genetic abnormalities [96]. CDH, for instance, allows abdominal organs to intrude into the thoracic cavity, limiting lung development [97]. Similarly, oligohydramnios, or reduced amniotic fluid, which can result from renal anomalies, prolonged rupture of membranes, or placental insufficiency, contributes to the underdevelopment of lungs by reducing the fluid volume that the fetus inhales and exhales during gestation, a critical factor in lung maturation [98]. Genetic disorders like trisomy 18 and 21 can also lead to pulmonary hypoplasia, underscoring the multifactorial etiology of the condition [93].

Pathophysiology of pulmonary hypoplasia centers on impairment in the typical stages of fetal lung development (embryonic, pseudo-glandular, canalicular, saccular, and alveolar) [99]. Interruptions or alterations in any of these stages can result in pulmonary hypoplasia [100]. For instance, an aberration during the pseudo glandular stage, which occurs between the 5th and 16th weeks of gestation and during which the primary bronchial tree is formed, could lead to a decreased number of airway divisions [101]. Similarly, disruptions during the canalicular stage (16th week to 26th week) or saccular stage (26th week to birth), responsible for the development of distal airways, vascularization, and surfactant production, may result in diminished alveolarization, vascular abnormalities, and surfactant deficiency, respectively, all of which are critical for neonatal lung function and can contribute to the manifestation of pulmonary hypoplasia [102].

Clinical Features and Diagnostic Criteria for Pulmonary Hypoplasia

Pulmonary hypoplasia encompasses a broad spectrum of clinical presentations, ranging from immediate neonatal death due to profound respiratory distress to the occurrence of recurrent respiratory infections later in life. Associated anomalies like renal agenesis, skeletal dysplasia, and cardiovascular malformations further compound the clinical picture [103]. Additionally, the infant may present with a barrel-shaped chest, nasal flaring, grunting, and cyanosis. Prenatal ultrasound may reveal oligohydramnios and a small thoracic cavity, and postnatal imaging modalities (e.g., chest X-ray, CT, MRI) can help determine decreased lung volume, airway anomalies, or associated abnormalities [104]. Definitive diagnosis often necessitates histological analysis, which can reveal fewer and larger alveoli, as well as reduced bronchial branching [105].

Management of Pulmonary Hypoplasia

Antenatal treatment for pulmonary hypoplasia often begins with the administration of corticosteroids for fetal lung maturation in fetuses over 24 weeks of gestation, especially in cases of preterm premature rupture of membranes. Concurrent use of antibiotics and tocolytics is also common. Some studies, such as those by Locatelli et al., suggest that serial amnioinfusion could decrease perinatal complications and prolong pregnancy in cases of premature rupture of membranes at <26 weeks [106]; however, the amnioinfusion in preterm premature rupture of membranes (AMIPROM) pilot study found no statistically significant differences in fetal or maternal outcomes [107,108]. For immediate post-partum respiratory support of infants, supplemental oxygen or extracorporeal membrane oxygenation (ECMO) may be administered, and there is limited evidence indicating potential benefits from inhaled nitric oxide [109].

CDH

CDH is a rare but serious birth defect where abnormal formation of the diaphragm occurs during fetal development. This abnormality allows for protrusion of the abdominal organs into the thoracic cavity, which can impede lung development, leading to life-threatening conditions such as pulmonary hypoplasia and pulmonary hypertension [97]. The defect typically arises on the left side of the diaphragm in (85% to 90% of cases) [110]. CDH affects approximately 1 in 2,500 to 5,000 live births worldwide, regardless of gender [111]. The exact etiology of CDH remains unclear; however, it is believed to be a complex interplay of genetic, environmental, and maternal factors. Most cases are sporadic, though some familial instances of CDH suggest a genetic predisposition, which is further supported by associations between CDH and various chromosomal abnormalities, copy number variations, and mutations in genes such as GATA4, ZFPM2, and FOG2 [111,112]. Environmental factors, including maternal smoking, alcohol consumption, and certain medications during pregnancy, also have been implicated as risk factors [113]. From a pathophysiological perspective, the herniation of abdominal organs into the thoracic cavity hampers normal lung development, leading to pulmonary hypoplasia. Additionally, the shift in organs and resulting pressure dynamics can lead to a significant increase in blood pressure within the lung's arteries (i.e., pulmonary hypertension), both of which are primary contributors to the high morbidity and mortality rates observed in infants with CDH [114].

Clinical Features and Diagnostic Criteria for CDH

CDH manifests clinically as severe respiratory distress shortly after birth due to the herniation of abdominal contents into the thoracic cavity, resulting in pulmonary hypoplasia and pulmonary hypertension. Physical examination may reveal abdominal breathing, cyanosis, and a scaphoid abdomen. Auscultation may highlight decreased or absent breath sounds on the affected side and displacement of heart sounds [115]. Antenatal ultrasound can detect CDH as early as the first trimester, though the second trimester is more common [116]. The lung-to-head ratio and observed-to-expected lung-to-head ratio can help predict disease severity and prognosis [117]. For the postnatal diagnosis, a chest X-ray may reveal elevated abdominal contents, and echocardiography can assess the presence and severity of pulmonary hypertension [116].

Management of CDH

In cases of CDH, fetal endoscopic tracheal occlusion can be employed to mitigate pulmonary arterial hypertension and hypoplasia, thereby increasing the chances of fetal survival. However, this technique can decrease the number of type 2 pneumocytes and, subsequently, surfactant proteins, which necessitates the deflation of the tracheal balloon shortly before delivery [118]. Surgical repair of such hernias should be delayed by 48-72 hours post-birth to allow for cardiopulmonary stabilization [119]. For patients on ECMO, a delayed approach can further reduce operative complications and enhance survival [120]. Adults who have survived pulmonary hypoplasia often contend with CLD, necessitating conservative management strategies such as the use of bronchodilators, antibiotics, chest physiotherapy, prophylactic vaccinations, and potential surgical resection in cases of localized bronchiectasis associated with recurring respiratory infections [120].

To treat CDH, it is crucial to conduct consistent monitoring to ensure the fetus’s well-being. Some U.S. medical centers offer fetal therapy for moderate to severe CDH, which involves occluding the fetal trachea with an inflated balloon, leading to an accumulation of lung fluid and thus promoting lung growth. However, this procedure can also reduce the number of type 2 pneumocytes, impacting surfactant production. Therefore, the balloon is typically removed around the 33rd or 34th week of gestation, although this procedure is associated with a higher risk of preterm delivery. Postnatal repair of CDH is always necessary following fetal surgery, and several ongoing multinational RCTs are evaluating the efficacy of tracheal occlusion in fetuses with moderate to severe CDH [121-123]. Regarding postnatal management, it is not advised to deliver infants with CDH before the 37th week of gestation, as studies show a significantly lower mortality rate in infants delivered at the 40th week [124]. Delivery at a tertiary center with expertise in CDH management and access to ECMO therapy is strongly recommended. Ventilator management is critical to prevent lung injury by maintaining gentle ventilation strategies [125]. Management of pulmonary hypertension, a common condition in infants with CDH, typically includes optimizing ventilatory settings, maintaining normal systemic arterial blood pressures, and using pulmonary vasodilators as necessary [125,126]. Surgical treatment of CDH has transitioned from an emergency procedure to a delayed surgery, 48 to 72 hours after birth, or even longer in severe PH cases. The approach to surgery also has evolved to incorporate minimally invasive techniques [127-129]. Despite this progress, it is recommended to continuously refer to standard practice guidelines for CDH [130-132].

CPAM

CPAM, previously known as congenital cystic adenomatoid malformation, is a rare lung condition marked by the development of cystic masses in the lung tissue during the fetal period [133,134]. These masses originate from anomalous embryonic development, resulting in air-filled cysts or solid lesions of varying sizes. The incidence of CPAM is reportedly 1 in 10,000-35,000 births [135]. Definitive causes of CPAM remain uncertain but are believed to be sporadic without a clear genetic predisposition [136]. Some researchers have proposed aberrant signaling during bronchial development as a contributor to the formation of these cysts [137]. Evidence thus far does not suggest a specific maternal or environmental factor. Most CPAM cases appear to occur randomly, with no discernible familial patterns or links to maternal lifestyle factors, such as smoking or alcohol consumption during pregnancy [138]. Regarding the pathophysiology, the abnormal air-filled cysts or solid lesions associated with CPAM can lead to compression of the surrounding healthy lung tissue, which can result in respiratory distress shortly after birth or later [139]. If the cysts become infected, they can cause severe lung inflammation and infection, leading to additional complications [140].

Clinical Features and Diagnostic Criteria of CPAM

CPAM may be asymptomatic, or symptoms may range from respiratory distress at birth to recurrent pulmonary infections or pneumothorax later in infancy [141]. Physical examination may reveal decreased breath sounds, crackles, and increased anteroposterior chest diameter. Prenatal ultrasound is commonly used for the detection and identification of cystic lesions of varying sizes. Further delineation of lesion characteristics and extent can be achieved via fetal MRI [105]. Postnatally, chest CT scans can provide a detailed view of the lung architecture, aiding in diagnosis. In certain cases, surgical resection of the lesion may be necessary, serving both diagnostic and therapeutic purposes [142].

Management of CPAM

When CPAM is detected prenatally, proactive management strategies can be employed, particularly when there is an associated risk for fetal hydrops. Clinicians might opt for interventions like fetal surgery, administration of corticosteroids, or drainage to avert fetal death [10]. Postnatally, surgical resection is the preferred management strategy for infants displaying symptoms of respiratory distress [143], as well as for those with lesions occupying more than a fifth of the hemithorax, those with bilateral or multifocal cysts, those with a pneumothorax in the context of CPAM, or those with a family history of pleuropulmonary blastoma. Even for older children with less severe symptoms, resection is commonly performed to preclude recurrent infections and potential malignancy risk, especially for known type 4 lesions [144]. However, for asymptomatic patients, the management strategy (i.e., elective resection or conservative observation) should be decided after discussing the benefits and drawbacks of each approach with the family and at the provider's discretion [145].

PIG

PIG is a rare lung disorder predominantly identified in infants and characterized by the accumulation of cells filled with glycogen in the lung's interstitial spaces [146]. The precise cause of PIG remains unclear; however, it is hypothesized to result from an arrest in the normal maturation of interstitial cells during lung development [147]. PIG has been associated with congenital heart diseases, suggesting a potential link between cardiac and pulmonary development [148]. Specific risk factors have not been definitively established, however, due to the rarity and relatively recent recognition of the disease. Evidence thus far does not suggest a genetic predisposition or environmental trigger for PIG, although further research in these areas is warranted [149]. In terms of pathophysiology, the accumulation of glycogen-filled cells within the lung's interstitial spaces can lead to thickening of the alveolar walls, potentially disrupting normal gas exchange and resulting in persistent hypoxemia. Over time, the persistent interstitial thickening can progress to fibrosis, leading to chronic respiratory insufficiency [150].

Clinical Features and Diagnostic Criteria of PIG

PIG is typically characterized by the presentation of tachypnea, retractions, and failure to thrive within the first few months of life, although symptoms can be non-specific. Physical examination may reveal signs of respiratory distress like tachypnea, intercostal retractions, and nasal flaring. Auscultation can reveal crackles or decreased breath sounds [151,152]. Chest radiography may show a diffuse interstitial pattern, and high-resolution CT may reveal ground-glass opacities. The definitive diagnosis of PIG, however, requires lung biopsy, displaying interstitial thickening with the accumulation of glycogen-laden cells [149].

Management of PIG

Management strategies for PIG are multifaceted and largely supportive, as there is no definitive cure at this time. In severe cases, management involves supplemental oxygen and mechanical ventilation [153]. Patients often require nutritional support due to the high caloric demands from the increased work of breathing. Corticosteroids have been tried in some cases with varying success, and lung transplantation remains a last resort for those with end-stage disease [154]. Future research is necessary to develop targeted therapies and better understand this rare pediatric disease.

NEHI

NEHI is a rare form of childhood interstitial lung disease, primarily observed in infants and young children [155]. NEHI is characterized by excessive proliferation of pulmonary neuroendocrine cells, particularly within the bronchioles, although the specific etiological cause remains unclear [156]. Current hypotheses propose that NEHI may result from an abnormal response to environmental triggers or viral infections in genetically predisposed individuals, though more research is needed to confirm these theories [157]. As with many rare pulmonary diseases, the risk factors associated with NEHI are not well understood. Epidemiologically, there appears to be no predisposition towards gender, but most cases of NEHI are diagnosed within the first two years of life [158]. Incidence rates suggest familial clustering in a small number of cases, hinting towards a possible genetic contribution to NEHI [156,159]. From a pathophysiological perspective, the hyperplasia of neuroendocrine cells in the lungs' small airways has been linked to airway obstruction, resulting in clinical manifestations such as tachypnea, hypoxemia, and failure to thrive. However, the precise mechanisms by which neuroendocrine cell proliferation causes these respiratory complications remain elusive and warrant further study [157].

Clinical Features and Diagnostic Criteria of NEHI

NEHI typically manifests within the first two years of life with symptoms such as persistent tachypnea, cough, hypoxemia, and failure to thrive. These children may have a normal physical examination, or there may be evidence of hypoxemia with digital clubbing or respiratory distress. Chest radiographs are often normal, but high-resolution CT scans may reveal ground-glass opacities primarily in the right middle lobe and lingula, referred to as the "mosaic pattern" [157]. The gold standard for diagnosis is a lung biopsy, which will show an increase in bombesin-positive neuroendocrine cells in the bronchioles. However, in the presence of characteristic clinical and radiological findings, biopsy may be avoided [158].

Management of NEHI

As a type of childhood interstitial lung disease, NEHI often requires supplemental oxygen therapy to maintain normal oxygen levels, especially during sleep or illness [160]. Pulmonologists might use bronchodilators to help improve airflow and steroids to reduce inflammation, though their effectiveness varies from patient to patient. Nutrition support can be crucial due to the increased energy needs from chronic hypoxia and the work of breathing. NEHI can present in diverse ways; thus, regular monitoring of lung function, growth, and development is necessary for optimal management [157].

ACD/MPV

ACD/MPV is a rare and severe developmental disorder affecting the formation of the lung's blood vessels and air sacs (alveoli) [161]. The exact cause of ACD/MPV is largely attributed to genetic mutations, predominantly in the FOXF1 gene, which plays a crucial role in lung development [162]. Most cases are sporadic, though a few familial occurrences suggest possible autosomal dominant inheritance with reduced penetrance [161]. Identifiable risk factors for ACD/MPV are currently limited, largely due to the genetic nature of the disease and its rarity. There is no known association with environmental factors, maternal health, or prenatal exposures [163,164]. Pathophysiologically, ACD/MPV is characterized by abnormal development and positioning of the lung's blood vessels, specifically the capillaries, which are crucial for oxygen exchange [165]. This misalignment and underdevelopment lead to a failure in the proper diffusion of oxygen from the alveoli into the bloodstream. Consequently, infants with ACD/MPV present with severe hypoxemia and respiratory distress shortly after birth, and the condition is often fatal within the first month of life [166].

Clinical Features and Diagnostic Criteria of ACD/MPV

ACD/MPV often presents in the immediate neonatal period with severe hypoxemia and pulmonary hypertension that is unresponsive to treatment. Clinical features might include cyanosis, severe respiratory distress, and hepatomegaly. The chest radiographs are non-specific, showing diffuse, hazy opacities or ground-glass appearance [167]. Echocardiography might reveal right ventricular hypertrophy and pulmonary hypertension. The definitive diagnosis of ACD/MPV is confirmed by histopathological evaluation of lung tissue, showing characteristic findings of misaligned pulmonary veins and a paucity of capillaries within the alveolar septa [168].

Management of ACD/MPV

The primary management strategy for ACD/MPV is supportive care, including mechanical ventilation and supplemental oxygen to assist with breathing difficulties; however, these interventions are usually insufficient to overcome the underlying pathology [163]. The use of inhaled nitric oxide, a potent pulmonary vasodilator, has been attempted but generally proves to be ineffective long-term due to the fundamental abnormal vascular development [169]. Lung transplantation has been suggested as a potential treatment, but the challenge lies in diagnosing this condition early enough and finding suitable donors [170].

Future directions

New treatment approaches have focused on the significant impact of intrauterine exposures on the development of both BPD and BPD-related pulmonary hypertension. For instance, administering vitamin C supplements to expectant mothers who smoke during pregnancy has demonstrated promise in enhancing pulmonary function tests and reducing wheezing incidence in newborns [171]. However, further research is required before implementing this approach widely. Another intervention involves administering N-acetylcysteine to pregnant women experiencing preterm labor with confirmed intrauterine infection or inflammation [172]. A phase I trial showed that this treatment significantly reduced BPD incidence in infants born to treated mothers, compared to those in the placebo-controlled group. Larger trials are needed to validate the effectiveness of this approach before integrating it into standard clinical practice. Exogenous surfactant is another potential therapy to reduce BPD outcomes by delivering budesonide directly to the airspaces. In an RCT involving 265 very-low-birth-weight infants, a combination of 0.25 mg/kg budesonide with surfactant was compared to surfactant alone. The intervention group exhibited a significant reduction in the primary outcome of death or BPD [173]. Currently, the ongoing budesonide in babies trial (NCT04545866) aims to assess the pulmonary and neurodevelopmental outcomes of infants who receive a combination of budesonide and surfactant versus surfactant alone.

Deficiencies in insulin-like growth factor 1 have been associated with the development of BPD [174]. A phase II trial has shown promising results in significantly reducing BPD among infants who received treatment with recombinant human insulin-like growth factor 1 combined with its binding protein (rhIGF-1/rhIGFBP-3) [175]. A larger RCT investigating the potential benefits of this treatment (NCT03253263) is ongoing.

Gene therapy, specifically the use of recombinant adenovirus expressing vascular endothelial growth factor, has shown promising results in animal models by promoting lung maturation and decreasing pulmonary hypertension [176]. Mesenchymal stem cell therapies have shown promise in managing various neonatal conditions, including BPD [177]. A phase II RCT explored the intratracheal administration of mesenchymal stem cells to preterm infants and demonstrated a decrease in severe BPD incidence, particularly in a specific subgroup of infants [178]. To further assess the safety and effectiveness of these treatments, larger multicenter trials and clinical studies are ongoing (e.g., NCT03392467), including one evaluating the intravenous administration of extracellular vesicles derived from bone marrow mesenchymal stem cells in preterm infants (NCT03857841).

Emerging treatments for these conditions are relatively unexplored due to their rarity. However, advancements in technology and gene therapy offer promising results for the development of targeted treatments [179]. In CDH, for example, advancements in prenatal imaging and fetal interventions have helped elucidate the condition’s impact on lung growth and development, offering novel insights into disease progression and prognosis [116]. In pulmonary hypoplasia and CDH, genetic studies in animal models have identified the Wnt signaling pathway as a potential mediator of lung development and maturation, underscoring its potential role in pulmonary hypoplasia [81]. Identification of associated genetic mutations in the GATA4 and FOG2 genes has further elucidated the pathogenesis of CDH [180].

Studies on CPAM, PIG, NEHI, and ACD/MPV have made progress in recognizing the genetic and molecular components of these rare conditions. For instance, the FOXF1 gene has been identified as a crucial factor in the pathogenesis of ACD/MPV [181]. In PIG, novel research efforts are attempting to delineate the underlying mechanisms of glycogen-filled cell accumulation [149]. In NEHI, advancements in high-resolution CT imaging have improved diagnostic accuracy to facilitate a better understanding of disease progression [157]. Similarly, identifying and understanding the specific genetic mutations in CPAM can help develop personalized therapeutic strategies, such as selective bronchial occlusion or prenatal surgical intervention [182]. As our understanding of the genetic and molecular basis of these diseases expands, targeted therapies may provide more effective, safer treatment options for these conditions [182].

## Conclusions

CLDs in neonates, including BPD, pulmonary hypoplasia, CDH, CPAM, PIG, NEHI, and ACD/MPV, present significant healthcare challenges. These diseases carry a substantial burden of morbidity and mortality and require comprehensive, individualized management strategies. Current therapeutic options mainly involve supportive care, and these diseases often require a multidisciplinary approach to manage effectively. Advances in genomics and molecular biology (e.g., gene therapy and stem cell treatments) show promise for the treatment and prevention of neonatal CLDs, allowing for a more tailored approach that accounts for individual genetic, biochemical, and lifestyle factors. Collectively, these precision medicine approaches offer potential diagnostic methods and therapeutic targets to improve outcomes for neonates affected by these severe pulmonary conditions.

Considering the important role of personalized medicine in the future management of CLDs in neonates, continued research is critical. Future efforts should be aimed at understanding the complex pathophysiology of these conditions, improving early diagnostic criteria, and developing targeted treatment modalities. Furthermore, multicentric and longitudinal studies are needed to better understand the long-term outcomes and develop preventative strategies for these diseases. By embracing a comprehensive, innovative, and patient-centered approach, we can enhance the quality of care and outcomes for this vulnerable population.
